# MOOC-based blended learning for knowledge translation capacity-building: A qualitative evaluative study

**DOI:** 10.1371/journal.pone.0297781

**Published:** 2024-02-09

**Authors:** Christian Dagenais, Aurélie Hot, Anne Bekelynck, Romane Villemin, Esther Mc Sween-Cadieux, Valéry Ridde

**Affiliations:** 1 Department of Psychology, Université de Montréal, Montreal, Quebec, Canada; 2 Programme PAC-CI, Abidjan, Côte d’Ivoire; 3 Department of Psychology, Université du Québec à Montreal, Montreal, Quebec, Canada; 4 Department of School and Social Adaptation Studies, Université de Sherbrooke, Sherbrooke, Quebec, Canada; 5 Université Paris Cité, IRD, Inserm, Ceped, Paris, France; 6 Institut de Santé et Développement, Université Cheikh Anta Diop, Dakar, Senegal; University of the Witwatersrand, SOUTH AFRICA

## Abstract

This qualitative study investigated the effectiveness of blended learning using MOOCs (massive open online courses) for capacity-building in knowledge translation (KT). The evaluation followed Kirkpatrick’s updated model. A total of 23 semi-directed interviews were conducted with participants working at a research centre in Côte d’Ivoire, with a first wave of interviews immediately post-training and a second wave after five months. Results showed that the training met learners’ needs, with both the content and teaching format being deemed appropriate. Learners reacted positively to face-to-face activities and affirmed the importance of coaching for putting learning into practice. Specific KT skills and principles appeared to have been acquired, such as a procedure for structuring the KT process and improved skills for communicating and presenting scientific knowledge. Five months after the training, encouraging changes were reported, but the sustainability of the new KT practices remained uncertain. KT capacity-building initiatives in low- and middle-income countries struggle to meet demand. Little is known about effective KT training in that context, and even less in non-anglophone countries. The study presented here contributes to the understanding of success factors from the learners’ standpoint.

## Introduction

Capacity-building initiatives in knowledge translation (KT) are struggling to meet worldwide demand, especially among low- and middle-income countries (LMICs) [1,2]. Researchers or those in charge of KT (in intermediary positions between research and practice) must develop skills to communicate research results effectively to those who can benefit from them [3]. To help meet this demand for KT training in francophone countries, the RENARD Research Team on Knowledge Translation and the French National Research Institute for Sustainable Development (IRD) worked in collaboration to launch a series of massive open online courses (MOOCs) and to document learning practices in different countries. This article focuses on the blended learning experience of a group of researchers in Côte d’Ivoire. MOOCs can be accessed for free by an unlimited number of learners anywhere in the world, but blended learning using MOOCs, or blended MOOCs, mixes online and face-to-face teaching formats [4]. The term “knowledge translation” (KT) used in this paper encompasses all steps of the research-to-action process, from the production of research to its use. This includes all efforts to promote research use, whether interactive or not [5].

### Effectiveness of MOOCs and learning contexts

Despite the positive potential of MOOCs, the high dropout rate of learners [6] remains a significant challenge. On average, only 13% or less of all enrollees complete their course [7–10]. The rate may be higher in a professional development context [11,12] or in vocational education and training (VET) [13]. Of those initially declaring an intention to complete the course, the average completion rate was 20% [14]. In addition, MOOCs have to date reached more people from high-income countries [15]. Research has traditionally focused on these “mainstream consumers” of MOOCs and has rarely addressed the learning contexts, motivations, and characteristics of learners in LMICs [16].

Although several strategies to combat high dropout rates have been proposed, few have been evaluated [9]. Nevertheless, learner engagement, such as the intention to complete an online course, emerges as an essential variable [16,17]. MOOC completion might not be the only indicator of success, however, given that individual training goals vary, as do strategies to achieve them [18–21].

Good-quality evaluative studies account for very little of the literature on MOOCs

[17,19,22,23]. Several MOOC quality criteria (technical, organizational, or social) are explored in the literature [24]. Evaluation models should therefore consider the diversity of these dimensions [16,17,25]. Furthermore, as experiments in blended learning with MOOCs increase [26], research should address the effects of diverse learning experiences on course completion, learning, retention, or application of new knowledge [16].

### MOOCs and KT training in LMICs

While there is little evidence on effective interventions to improve KT skills in LMICs [27,28], many authors describe best practices to be adopted. Regardless of the instructional format, understanding the diverse profiles of individuals or organizations and their needs in this area is crucial [28,29]. It is also important to foster team-based learning supported by strong collaborations [30], impart the knowledge and skills needed to engage communities and decision-makers, and translate research findings into practical recommendations [29]. Workshops that offer hands-on learning, coupled with mentoring opportunities, may be a valuable choice for training [31].

As for MOOC-based training for health workers in LMICs, a recent literature review identified the blended learning mode as a factor facilitating completion and assimilation of content [32]. A MOOC designed to improve the implementation science skills of scientists and professionals in this context improved practices [11,33].

For a better understanding of the potential of MOOCs for KT training in LMICs, diverse learning experiences and experiments with blended formats should be investigated. This article aims to contribute to this knowledge base with a qualitative evaluative study.

## Materials and methods

### Description of the learning experience under study

This article describes the blended learning experience of researchers at the PAC-CI Program, a research centre in Côte d’Ivoire. The PAC-CI Program is internationally recognized for its work in clinical research. It is currently diversifying disciplines and research topics (non-communicable diseases, sexual and reproductive health and rights, etc.) and moving towards greater recognition and impact on a national scale and in the field. PAC-CI was awarded a one-year technical support grant from L’Initiative/Expertise France, with a twofold objective of providing KT training and developing KT activities and tools, including 11 policy briefs.

As a mandatory prerequisite to face-to-face training, participants completed a first MOOC, "Introduction to Knowledge Translation," which consists of eight modules for an estimated 20 hours of learning. These modules cover the definition, principles, issues, and evaluation of KT and some translation tools or activities. CD (distance instructor) and AB (national researcher and organizational spokesperson) organized two videoconference sessions: one at the beginning of the learning process and another midway through the MOOC. These sessions provided opportunities to ask questions live while setting a learning pace.

Subsequently, a one-week face-to-face training session focused on a second MOOC, "Preparing a Policy Brief." Five modules, for an estimated 15 hours of learning, cover the definition and characteristics of policy briefs, the state of research on this KT tool, and the practical steps for developing and disseminating policy briefs. Eighteen members of the organization attended this training, which was primarily designed to build practical knowledge. The face-to-face training alternated between instructional videos, quizzes, and small group activities to draft four policy briefs.

**Fig 1:**
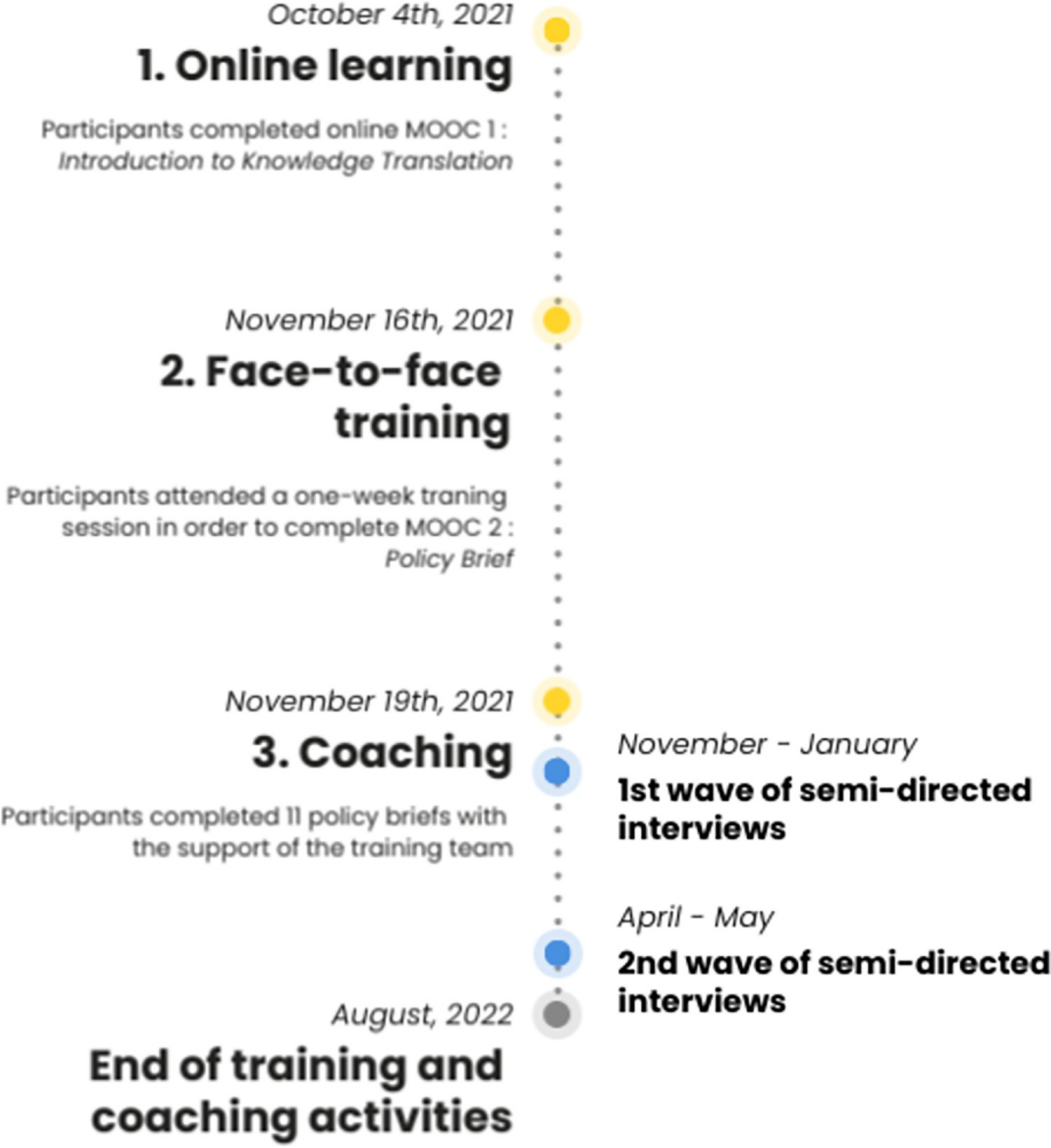
Training and evaluation schedule.

In the weeks and months following, learners were coached by AB, CD, and AH (distance trainer), both in-person and remotely, to finalize policy briefs or develop other KT tools and activities (KT plans, oral presentations, workshops). All activities were conducted in French. For the schedule of activities, see Fig 1.

### Theoretical framework

As in previous MOOC studies [6,11,34], the evaluation of the blended training followed Kirkpatrick’s updated model [35]. This internationally recognized model measures the effectiveness of all types of training to improve professional practice. It includes four levels of evaluation. The first level aims to evaluate **participants’ reactions** to training, including their involvement, satisfaction level, and the relevance of course content. The second level seeks to assess **participants’ learning** following training (knowledge, skills, attitudes, confidence, intention), and the third aims to evaluate **behaviour changes**, i.e., the application of new knowledge or skills as well as the facilitators or obstacles to these changes. Finally, the fourth level evaluates **the impacts of these changes**, i.e., the potential impacts of KT activities carried out by learners.

### Method

Using a qualitative design aimed at documenting learning experiences [36], the study’s objective was to gain a more in-depth understanding of the learners’ profiles, the learning context, their experiences with the blended format and, finally, their use of the knowledge acquired.

Data were collected in two waves of post-training interviews targeting all trainees: 18 persons working at PAC-CI. The evaluation team (AB, AH, CD) also documented all activities carried out following the training (deployment of KT strategies), as well as their own coaching experience.

A first wave of interviews with 12 respondents following the face-to-face training (November 2021 to January 2022) explored levels 1, 2, and 3 of Kirkpatrick’s model: reactions, learning (including the intention to apply learning), and behaviour change. The interviews also examined processes, including facilitators and obstacles in terms of technological, pedagogical, individual, and contextual factors, drawing on the TIPEC conceptual framework [37], and how these processes were affected by the different formats of the two MOOCs. The interview grid for the first wave (S1 File) addressed these different themes. Interviews lasted between 45 and 75 minutes and were conducted via the ZOOM videoconferencing platform. Recruitment of new respondents stopped in accordance with the principle of saturation, i.e., when no new ideas emerged with additional interviewees [38].

The second wave of interviews targeted the 12 respondents of the first wave, of whom 11 accepted to participate. This second wave aimed to evaluate the sustainability of the learning and its effects (use) five months post-training. The interviews also explored ways to improve training and coaching, as well as avenues for overcoming implementation issues. The interview grid (S2 File) mainly included questions on knowledge application—including participants’ revisiting of course materials online—and on the perceived impacts of the MOOCs at the individual and organizational levels. Follow-up interviews lasted between 11 and 28 minutes and followed the abovementioned method.

The interviews were recorded, partially transcribed, and analyzed using NVivo R.1^©^ qualitative analysis software. Partial transcription was done to segment the contents of the audio recordings and transcribe main ideas, and complete quotations for the most relevant passages were transcribed as needed. Segments remained linked to the audio content and were available for repeat listening throughout the analysis. A thematic analysis focused on each learner’s profile (professional characteristics, learning habits, etc.), their reactions to the learning experience, their learning, and the behaviour changes they observed (including facilitators and obstacles to change). The fourth level of Kirkpatrick’s model, on the impacts of observed changes, was addressed more specifically through content analysis of the follow-up interviews.

Learning experiences, as described in the interviews, were compared to training experiences (activities carried out, issues, facilitating factors and obstacles), as documented in various progress reports and discussions among research team members. This comparison enriched the interpretation of results.

### Ethical approval

This study obtained ethical approval from the Université de Montréal’s Educational and Psychological Research Ethics Committee (CEREP-21-087-D). Informed consent was obtained in writing from the participants prior to data collection.

## Results

Fourteen of the 18 people trained completed MOOC 1, with an average grade of 76%, and 11 completed MOOC 2, again with an average of 76%. Of these 18 people, 12 agreed to participate in the first wave of interviews, three women and nine men. They obtained a 79% average for MOOC 1 and a 75% average for MOOC 2, although two dropped out of the second MOOC. Eleven of these respondents, three women and eight men, participated in a follow-up interview (–1 compared to the first wave).

### Learner profiles

The participants held various positions in their organization, including junior and senior researchers, pharmacists, doctors, social scientists, and managers. Despite this diversity, most were accustomed to taking and even offering online courses, even more so since the COVID-19 pandemic. They appreciated the choice and access that online courses represented, but for many, it was a choice they fell back on by default in the absence of face-to-face courses.

“Yes, with the advent of the Coronavirus, we ultimately got used to them [online courses], but I’d say I prefer face-to-face courses because several aspects are relevant for me when taking a course… Having the instructor there, being able to discern certain emotions, well, how can I put it, there’s a communication that’s not necessarily verbal that also matters to me, that also helps me understand, it’s a whole package that I value.” [04COTACC]

In addition, most participants reported prior experience with KT activities (e.g., feedback workshops, oral presentations), which was not surprising, given that they were part of an active research community.

Regarding the blended format, many appreciated the opportunity to take the first MOOC, which was more theoretical, alone, and the second in a group as they moved towards practice. Although the participants considered the online course easy to follow, having the time to go deeper into the course during in-person discussions and being able to ask questions helped them to integrate the content much better. Participants reported that certain grey areas in the learning process would persist if people went through the MOOC alone without opportunities for discussion, especially on the answers to the module exams.

“When we watched the videos, we could ask lots of questions, so that was the added value for me, to facilitate discussions… For example, one of our seniors listened to everything, he thought he had understood, and he only asked for the quizzes to test whether he had understood or not, and in fact, with the quizzes, he realized that it wasn’t easy to listen and understand everything… .” [08COTACC]

On the other hand, the face-to-face training was intense. It was spread over five half-days in one week, which required a level of concentration that, for some, had more impact on their behaviour than fragmented online learning. People were also able to get together for the first time since start of the pandemic, which increased motivation.

Finally, with face-to-face training, they could share experiences with people from different disciplines, think more deeply about how to apply the knowledge, and acquire know-how.

“For something like knowledge translation, I think it’s really worthwhile for [the training] to actually be face-to-face, because there’s a know-how that’s not just technical… But [for KT training], there’s this really useful aspect of discussing experiences and things that were done, which are really difficult to convey fully online.” [05COTACC]

Given their familiarity with online courses and their perseverance or discipline, in their own words, most believed they would have completed MOOC 1, “Introduction to Knowledge Translation,” even without coaching from the training team. Nonetheless, others indicated that the distance coaching was helpful, especially given the tight timeframe imposed by the training schedule. For the second MOOC (“Preparing Policy Briefs”), opinions were more divided; many felt that acquiring practical knowledge requires closer supervision and coaching.

### Learning

Participants reported a significant difference in their KT knowledge pre- and post-training. Nevertheless, during the first wave of interviews, i.e., immediately after training, one-third of participants emphasized that practical experiences were essential to make this learning concrete.

With respect to attitudinal change, they described a “paradigm shift” with a strong emphasis on KT since taking the course and noted the idea that the usual practices—feedback workshops and scientific publications—were no longer sufficient.

“So, after almost 20 years in this field, I’m happy to have heard about knowledge translation, and when you find out about it, you realize there’s another way of communicating research results to users. I wasn’t exaggerating when I said that for us, for me, it’s a pretty significant change in practice, a paradigm shift… it’s really important.” [02COTACC]

Several participants referred to acquiring a methodology: “…in the beginning, we did it ‘like that’, with no methodology, with no structure” [12COTACC] or any know-how for carrying out the KT process. In the training, they were able to go through the different stages for preparing the policy brief: “I know how to present this policy brief to convince decision-makers… obviously, to solve a public health problem” [09COTACC]. Beyond policy briefs, other tools were also of interest to the trainees:

“Even in the MOOC, they explained to us how to make videos, how to prepare an oral communication, how to present [things], it was really enriching, how to make infographics. It’s true that the MOOC didn’t go into depth, but we know, for example, that we can find software that can help us… and that there’s a way to get the message across.” [11COTACC]

The participants also noted that they had acquired new science communication skills tailored to target audiences. More broadly, one trainee felt that her writing skills had improved:

“In terms of my writing style, now I try to get to the point while providing the very essence of the idea, in fact. Before, it used to be about writing [many] pages and displaying the extent of your knowledge in your discipline, but now it’s about… everyone being able to understand what I’m producing. The intention is for the data to be correctly grasped and understood.” [04COTACC]

One trainee involved in KT activities also described acquiring the soft skills of being available and accessible to support the target audiences properly and being flexible in adapting the language and format to those audiences.

The intention to use the knowledge was expressed in different ways: revisiting the course materials, implementing a KT strategy, developing KT tools, and presenting results in different ways, whether for funding applications or with a view to becoming a knowledge broker.

### Facilitators and obstacles

The participants noted several factors that facilitated learning and implementation and enabled them to carry out the planned work successfully: organizational support with management encouragement; the presence of a dynamic organizational spokesperson (AB) who was primarily involved in implementing activities; and finally, the support of an experienced instructor (CD). Group dynamics in the classroom, professional motivation (to obtain a certificate, especially among junior researchers), and the trainees’ intrinsic motivation were also facilitating factors.

“…knowledge translation is important, because these are concepts that aren’t sufficiently well known here, and when you write in your CV that you had to do a MOOC on knowledge translation that was validated, with both face-to-face and distance learning, automatically it whets the curiosity of the recruiter or the person who wants to offer you a consultancy or a job… so, already, that will distinguish me, the doctoral student, from the other [candidates].” [06COTACC]

As for factors that supported actual use of the learning, respondents said that engaging in writing policy briefs as a group motivated them to keep going. Coaching and evaluation activities that spanned several months also helped keep the trainees motivated.

Among the factors that participants identified as hindering their application of KT knowledge were lack of time and the organization’s busy schedule of activities, i.e., “falling back into a routine” that did not include KT activities. Also, some projects were not yet at a suitable stage for KT. Finally, resistance to change, attachment to habitual thinking, and peer group jargon hindered adherence to KT principles.

### Observed changes and potential impact of training

Regarding reported changes, preparing policy briefs represented the bulk of their application of new knowledge, with most participants having been involved in that work to varying degrees (leaders, contributors, etc.). Four people indicated that they used the knowledge they acquired for knowledge-sharing activities (workshops), developing presentations (including slides) based on the principles and tools seen during the training (synthesizing, using simple language), and writing funding applications (for research projects or consulting).

From an organizational standpoint, according to some trainees, the MOOCs contributed to an organizational transformation; they had a leveraging effect on this transformation and were a gateway to innovation. The KT training helped them to transition from international recognition and excellence to local or national legitimacy.

"We [PAC-CI] are becoming increasingly better positioned, because it must be said that for some time, after all,… . But we’ve evolved a lot… Because we had results that, in the beginning, we didn’t necessarily share locally; we published a lot in international journals, and all that. Then as time went by, we started to organize what we called our scientific days, and these were occasions where we opened up more and more, so we opened up to not only the local, national scientific community but also to other people, or patients—in fact, patients, because we work with patient organizations, civil society, journalists, and all that. Yes, PAC-CI can position itself as a knowledge translation tool.” [12COTACC]

Others felt that KT practices at PAC-CI had not changed significantly beyond the planned training and coaching activities (development of policy briefs, planned deliberative workshops). They believed it was “a little early to tell” [06COTACC] whether the training had changed practices. Although it was not yet clear whether the KT dynamic would be sustainable, some noted that KT was now part of the conversation in the organization, particularly in anticipating what a project’s results would be and how to promote them. KT was also a follow-up item on the agenda of scientific management meetings: “We make observations objectively. We try to find appropriate solutions to move forward” [02COTACC]. Others pointed out more subtle changes at the individual level, particularly in scientific communications at conferences:

“[In their communication], they shared information that was really essential, given that the audience that was going to receive the information wasn’t from the medical sciences, and it was information that was presented in a way that everyone could understand. I think the training had an impact on PAC-CI through these researchers in their way of communicating.” [06COTACC]

The participants suggested recruiting a dedicated knowledge coordinator or broker to embed KT into sustainable organizational practices, cultivate local networks, and overcome the lack of time and the resistance to conducting KT activities. They also recommended ensuring funding for KT activities by including this line item in future research grant applications.

The main results are presented in Table 1.

**Table 1:** Main results according to Kirkpatrick’s model.

REACTIONS	LEARNING	BEHAVIOUR CHANGES	POTENTIAL IMPACT
• Training met a need • Appropriate learning process, from theory to practice • MOOCs easy to follow • Relevant content in both MOOCs • Blended format suited to learning context • Beneficial face-to-face training: e.g., better understanding, know-how acquisition, and group learning dynamics	• Attitude change toward KT • Acquisition of a KT method and structure • Skills for writing policy briefs • Knowledge of other KT tools • Writing skills adapted to the target audience • Know-how acquisition • Soft skills • Intentions to use (e.g., revisiting course materials, implementing KT strategy, developing tools, presenting results in different ways, becoming a knowledge broker)	• Production of policy briefs • Following KT principles while participating in workshops • Following KT principles for oral communications and slides • Writing research grants or consultancy proposals (content and format influenced by KT training) Barriers to implementation: • Not enough opportunities for practice or too little coaching • Not enough time, heavy workload • Unfavourable timing of projects • Resistance to change on the part of researchers (habits) Facilitating factors: • Management support • Commitment of a resource person (spokesperson) • Coaching and evaluation process • Professional, intrinsic, or group motivation	• Individual level: participants follow KT principles and use some of the new skills acquired • Organizational level: commitment of management to sustain the implementation of KT strategies

## Discussion

The aim of this evaluation was to assess the potential impact of blended MOOCs for capacity-building in KT. The training met learners’ need, with both the content and teaching format deemed appropriate. Trainees’ reactions to face-to-face activities were positive and reiterated the importance of coaching to put learning into practice. Specific KT skills and principles seemed to have been acquired, such as a procedure for structuring the KT process and better skills for communicating and presenting scientific knowledge. Several months after the training, encouraging changes were observed, pointing to a longer-term benefit: effective use of KT skills and the acquisition of know-how by many, as well as a formal organizational commitment to supporting new KT practices.

In the context of this study, one success factor of note was the trainees’ profile. They were members of an active scientific community familiar with online training who adopted good self-regulation strategies from the get-go [16,39]. Unlike other evaluative studies in the context of LMICs [27], the results also revealed changes in people’s attitudes towards KT. The structured, time-bound blended format facilitated individuals’ learning engagement and content assimilation [32]. Few participants dropped out, due to this team-based organization-wide learning environment [16,29]. Peer-to-peer motivation, stimulated by the week of in-person classes and contextualized coaching, also promoted learning [40]. Beyond cognitive motivations, many participants expressed a social and emotional engagement in the training activities offered at a specific time during the COVID-19 pandemic. Finally, engagement and motivation remained relatively high as participants focused on small successive goals [41], such as finishing modules in the allotted time or contributing to the preparation of policy briefs.

Despite this overall positive learning experience, the sustainability of implementation efforts remained partially dependent on organizational supports, such as the availability of a dedicated person within the organization and, more broadly, the necessary resources and opportunities to put the acquired knowledge into practice [33]. This reiterates the need, when embarking on any such initiative, to consider capacity-building at not only the individual level, but also the organizational level [28] so that KT activities become embedded in organizational routines. Indeed, the sustainability of innovations or programs may be assessed through four characteristics of organizational routines: memory, adaptation, values, and rules [42]. Memory refers to past experiences within an organization that members can share. It requires stable resources dedicated to sustaining the implementation of a program, which, in the case under study, were not yet secured. The trainees could count on a fair amount of support from management and the training team (including a local spokesperson) for several months to develop concrete KT tools [1]. As in similar KT capacity-building projects, this coaching phase appeared crucial for gaining practical knowledge [31] and adapting the training to the local context. Management support was experienced when KT activities and strategies were integrated into organizational routines as expressions of the organization’s values. Nevertheless, at the time of the second round of interviews, there were as yet no explicit organizational rules about integrating KT activities into future projects.

While large cohorts can take a MOOC independently, only smaller groups can receive coaching, as described here, which limits the number of people targeted [1]. Thus, it would appear essential, in future research, to continue to measure the added value of coaching compared to other ways of supporting the application of the learning in the medium and longer terms. As for future capacity-building initiatives, organizations and training teams should consider that learners need ongoing coaching support past the active training phase. Organizational commitment to sustaining KT practices is crucial, as would be the specific training of individuals who could take on the role of knowledge brokers and support internal organizational capacity-building.

### Limitations

The data collected in this study do not allow for generalization. They represent a particular case and shed light on the success factors for other KT training or blended format activities. In addition, this research used a qualitative approach to report on learning experiences. Therefore, the evaluation model (Kirkpatrick model) was not used to its full potential since, for example, the learning was not subjected to a pre–post quantitative evaluation.

It should be noted that the interviewees had completed at least one of the two MOOCs. Targeting trainees who dropped out of both courses might have brought to light other obstacles to learning. Finally, a social desirability bias cannot be ruled out, as the interviewees may have wanted to present their learning experience positively.

## Conclusion

This article presents the results of an evaluation that followed learners over several months to assess the potential impact of a blended learning experience in KT. Describing this learning experience from the trainees’ perspective highlighted critical motivational factors, including professional context, group motivation, and appropriate support in carrying out practical post-training tasks. The face-to-face component of the training and active contribution to KT activities were necessary to acquire know-how. The evaluation of similar capacity-building activities, especially in KT, should focus on the added value of face-to-face training or coaching measures compared to other potentially effective measures to acquire know-how, which could be implemented on a larger scale.

## Supporting information

S1 File Interview grid (1^st^ wave). (DOCX)

S2 File Interview grid (2^nd^ wave). (DOCX)
