# Supplementary material for: MOOC-based blended learning for knowledge translation capacity-building: A qualitative evaluative study
Source: PLoS One. 2024 Feb 9;19(2):e0297781. doi: 10.1371/journal.pone.0297781 (PMC10857586; doi:10.1371/journal.pone.0297781)
Supplement: S1 File — (DOCX) [file pone.0297781.s001.docx]

**INTERVIEW GRID (post-MOOC)**

Evaluation of the series of MOOCs on knowledge translation and modes of participation

"Before we begin the interview, I'd like to remind you of the objectives of this study. The aim is to assess the quality, usefulness and relevance of the MOOCs on knowledge translation in which you have participated, as well as the effects of two modes of course participation. Some participants followed the MOOCs on their own, while others were accompanied by an instructor.

Your participation is therefore solicited in order to better understand your learning experience and the potential effects of the series of MOOCs on knowledge translation. These courses were developed by the RENARD research team headed by Université de Montréal researcher Christian Dagenais.

It's thanks to your feedback that we'll be able to better understand how to improve the courses and better support learners. So, in this interview, I'm going to ask you a few questions on various themes surrounding your learning experience."

**Confidentiality of information collected during the interview:**

- Any information provided will be kept confidential.
- Any information provided will be processed and presented́ anonymously.
- t any time, you may discontinue your participation without any justification.
- Do you agree to the interview being audio recorded?

*Do you have any questions before we begin?*

**Context/contextual factors**

1. Did you follow the course alone or with an instructor?
2. What is your current job?
3. Could you briefly describe your organization and its missions?
4. How important is knowledge translation to you? Why or why not?

**Technological factors/accessibility**

5. What did you use to access the course? (Laptop, tablet...)

- Would you have preferred to access it via your smartphone?
- Was the content compatible with the computer you used? For example, was your computer or its system recent?

6. Did you encounter any difficulties connecting? Or any other technical/technological difficulties?

- Do you have a good Internet connection?
- Did this hinder your experience?

7. If you followed the course with an instructor: did you have any difficulty getting in and staying in touch with your instructor?

8. Was the course interface user-friendly? What would you change (font size, colors, video/image quality)?

- Was the course structure well organized and easy to follow? If not, what would you change?

9. Did you use the discussion forum? Was it relevant? Why or why not?

**Individual factors**

1. Did you already have some basic knowledge related to the course?

11. Why did you take this course? What were your motivations?

12. Are you comfortable with basic computer tasks?

13. Do you enjoy taking online courses?

14. Did you find the course easy to use?

15. [If you took the course alone] Was the asynchronous mode relevant? Or did the lack of interaction upset you?

- With an instructor
- With other learners

16. [If you took the course with an instructor] Did you use the opportunities for interaction offered to you? Was the presence of an instructor relevant?

- What did you appreciate most about the opportunity to interact with an instructor?
- If you had been able to have à la carte coaching, what would you have changed?
- Do you think you would have completed the course even without coaching?

17. Did taking this course cause any conflicts in your schedule? How did you organize your time (work/family/study)?

- During the e-learning sessions, were you in a supportive, distraction-free environment?
- Did you have the support of your family and friends or your employer?

18. What are your expectations for a future course like this?

**Pedagogical factors and content assessment**

19. Depending on your lifestyle, the concerns in your network or society, or your culture, does the course lack personalization/adaptability of content?

- Is the language adapted?
- Did the course content seem at times to be far removed from your daily concerns, or did any elements upset you? (Based on your beliefs, or values...)

20. What did you think of the exams? Were they too difficult?

21. Was the content relevant?

22. Were the materials provided varied enough? Of good quality?

- Which activity(ies) did you prefer (educational videos, quizzes, readings, practical exercises, Vox pop, summary sheets)? Why or why not?
- Which activity(ies) did you enjoy least? Why or why not?

23. What do you think would be the best way to get learners involved in such activities? [If you took the course with an instructor] Do you think that coaching is a good way of encouraging engagement?

**Application of knowledge and potential impact of the MOOC**

24. Do you plan to refer to and re-use your notes and the summary sheets provided during this course?

- 25. What did you retain from these presentations?

26. How would you describe your knowledge of knowledge translation before taking this course? What about now?

- What impact has this training had in the short and medium term?

27. In your opinion, how useful is this training for your practice? Are the recommendations made during the course adapted and applicable to your environment? Could you give me an example?

- Has anything changed in your practice since the course?
- Do you have or plan to apply any of what you learned during the course? How?

28. What are the obstacles to changing practices? (Lack of knowledge, lack of skills, lack of time, lack of resources, etc.)

29. Would you recommend this course to others? Why or why not? [If you took the course with an instructor], would you recommend another person to take the course alone or accompanied by an instructor? Why or why not?

**Other comments**

1. Do you have any other comments? Is there anything else you'd like to talk about?

- We plan to conduct follow-up interviews in six months.

**Thank you for your cooperation!**
