# Supplementary material for: MOOC-based blended learning for knowledge translation capacity-building: A qualitative evaluative study
Source: PLoS One. 2024 Feb 9;19(2):e0297781. doi: 10.1371/journal.pone.0297781 (PMC10857586; doi:10.1371/journal.pone.0297781)
Supplement: S2 File — (DOCX) [file pone.0297781.s002.docx]

**INTERVIEW GRID (Follow-up 6-12 months after course completion)**

Evaluation of the series of MOOCs on knowledge translation and modes of participation

"Before we begin the interview, I'd like to remind you of the objectives of this study. The aim is to assess the quality, usefulness and relevance of the MOOCs on knowledge translation in which you have participated, as well as the effects of two modes of course participation. Some participants followed the MOOCs on their own, while others were accompanied by an instructor.

Your participation is therefore solicited in order to better understand your learning experience and the potential effects of the series of MOOCs on knowledge translation. These courses were developed by the RENARD research team headed by Université de Montréal researcher Christian Dagenais.

It's thanks to your feedback that we'll be able to better understand how to improve the courses and better support learners. So in this interview, I'm going to ask you a few questions on various themes surrounding your learning experience."

**Confidentiality of information collected during the interview:**

- Any information provided will be kept confidential.
- Any information provided will be processed and presented́ anonymously.
- t any time, you may discontinue your participation without any justification.
- Do you agree to the interview being audio recorded?

*Do you have any questions before we begin?*

**Application of knowledge and perceived impact of the MOOCs**

1. Since the end of the course, have you returned to the ÉDUlib platform to look for tools, references or to view certain content again?

- Follow-up question: For what purpose?

2. Since the end of the course, have you referred to your notes and the summary sheets made available during the course?

- Follow-up question: have you used them? In what context?

3. Since our last conversation, have you worked on any KT tools or strategies? Which ones?

4. [If yes] What factors have helped you put your knowledge of KT into practice?

5. What factors have hindered you from putting your knowledge of KT into practice?

6. What are you missing in order to use the knowledge you have acquired about KT?

- Follow-up question: Do you think you've forgotten much of what you've learned through lack of use?

7. Have any of PAC-CI's KT practices changed since the training?

8. [If the respondent was accompanied by an instructor] Since the end of the course, have you been in contact with your instructor or other participants in your group about the knowledge and skills you have acquired? For what purpose?

9. Do you have any other examples of the usefulness of this course?

- Follow-up question: In your professional life? In your studies? In your job search?

**Other comments**

10. Do you have any other comments? Is there anything else you'd like to talk about?

- Thank you for your cooperation!
